# Travel demand and distance analysis for free-floating car sharing based on deep learning method

**DOI:** 10.1371/journal.pone.0223973

**Published:** 2019-10-16

**Authors:** Chen Zhang, Jie He, Ziyang Liu, Lu Xing, Yinhai Wang

**Affiliations:** 1 School of Transportation, Southeast University, Dongnandaxuelu, Nanjing, P.R. China; 2 Smart Transportation Applications and Research Laboratory, Department of Civil and Environmental Engineering, University of Washington, Seattle, WA, United States of America; Central South University, CHINA

## Abstract

In order to address the time pattern problems in free-floating car sharing, in this paper, the authors offer a comprehensive time-series method based on deep learning theory. According to car2go booking record data in Seattle area. Firstly, influence of time and location on the free-floating car-sharing usage pattern is analyzed, which reveals an apparent doublet pattern for time and dependence usage amount on population. Then, on the basis of the long-short-term memory recurrent neural network (LSTM-RNN), hourly variation in short-term traffic characteristics including travel demand and travel distance are modeled. The results were also compared with other different statistical models, such as support vector regression (SVR), Autoregressive Integrated Moving Average model (ARIMA), single and second exponential smoothing. It showed that (LSTM-RNN) shows better performance in terms of statistical analysis and tendency precision based on limited data sample.

## Introduction

Car sharing, as a fascinating travel mode, is now attracting global interest. Although it was first introduced the 1940s [1], not until recently, has car sharing become popular in North America. With the help of modern communication technology such as mobile apps that allow users to track location of available vehicles.

In general, there are several different modes for sharing of cars based on different operating strategies. Typical sharing modes include round-trip and one-way use. Round-trip car sharing usually required users to return vehicles to the same station, which causes inconvenience. In comparison, one-way car sharing is more flexible as the user is not required to return the vehicle to the original location; the user either returns the vehicle to a station at the destination or even leaves it parked on the roadside. The latter mode is usually called free-floating car sharing (FFT), which is analyzed in this paper. The first FFT was launched in North America in Austin, Texas operated by car2go, which is now the largest FFT company worldwide [2]. Car2go offers service in many cities around United States. In consideration of the authors’ studies in Seattle, related data from car2go in Seattle was chosen as the research source.

One concern in this research is description of the actual time variation in demand of FFT service; previous studies seem to pay more attention on the large-scale demand and pattern problem [1], but concern with the micro-scale time interval was rarely involved. Thus, in order to take full advantage of the booking records obtained from the car2go API portal (car2go API), the details of the time distribution will be analyzed to established a feasible method for short-term prediction. More detailed attention will be paid to parameters such as the travel demand and travel distance.

In short, based on the need of an appropriate processing for time-interval data, the present paper is aimed at an effective analysis method for real-time data. Firstly, a brief description of data collection is offered. Secondly, a model is established for microscopic prediction. At last, a comparison part is included for validation of the proposed model. It is expected that these efforts could provide some assistance for policy makers and FFT companies to facilitate such a travel model in a city.

## Literature overview

One-way FFT analysis started about 10 years ago, and is generally targeted at developed research cities in Germany and Switzerland [1–3], these cities are more susceptible to novel travel methods besides private vehicles and public transportation. Although these researches make some progress in the analysis of user patterns and demand prediction, their result display more dependence on large-scale surveys or national household surveys.

Former researches including: through a survey of over 1000 FFT members and over 1600 station-based car-sharing members, it was concluded that car-sharing members are influenced by other factors than car-sharing activity [1]. Furthermore, through exploring membership in different car-sharing services, the reduction in vehicle ownership after members joined car-sharing services was estimated, and the results showed that the proportion for members of Modo car sharing company, who gave up car ownership, were nearly 2 times larger than that for car2go users [4]. In addition, by two different methods in a survey from 1881 respondents, the impact of the car-sharing system car2go on other transport modes in Ulm, Germany was measured [5].

From the above research, it is certain that, although they made an effort to understand the demand, user patterns, and other macro-level indexes, few studies actually focused on the real-time data and short-term prediction for hourly variation, despite the fact that there existed some insightful researches for other traffic mode and large scale traffic demand analysis [6]. For example, taxi trips data was employed to infer and predict the human mobility and driving trajectories [7–10]. And methods such as adaptive neural network, Copula-Based approach were employed to understand microscopic vehicle lane changing and crash problem [11–13]. It was occasionally mentioned briefly in some FFT studies but their primary attention was laid on traditional relocation or spatial analysis [14–15], and do not significantly contributed to the short-term prediction problem.

According to the previous knowledge, several time-series methods will be considered as well. Among these, ARIMA is the most widely used. According to success from some previous researches on other transportation problems, the potential of this methodology is obviously of potential value [16–18] compared to other time-series methods. However, when running ARIMA, several key parameters should be precisely specified such as the auto-regressive parameter (p), the integrated parameter (d), and the moving average parameter (q). All these parameters are obtained by means of intensive fine-tuning process, and additionally a more periodical data set are required [19–21].

Thus by means of artificial intelligence, the model fitting and prediction problem could be improved in terms of time cost and accuracy based on a refined artificial neural network–recurrent neural network. Unlike the traditional neural network, it processes data sequences by storing a time-state containing information related to the former time condition. However, because of the major issue of dropping the long-term dependencies while in a deep net, RNN was evolved into the Long Short-Term Memory RNN which was first proposed in the mid-1990s [22–23]. They are trying to solve the gradient vanishing problem in the process of training the net. This model was validated to be an appropriate tool of addressing the time-series predicting problem in speech processing, word translation and recognition, transportation problems [24–28].

The present paper offers a comprehensive insight of free-floating car-sharing usage patterns within the context of location and time of different scale; then, the prediction model using the LSTM-RNN is employed to address the demand and travel distance prediction problem. Both models are based on hourly data set. Finally, a comparison between several models is made.

The rest of the paper is organized as follows. Section 3 describes the data process in detail, and then Section 4 offers a detailed process of constructing the LSTM-RNN model. In addition, the travel distance and demand analysis are also provided based on hourly data set. Section 5 comprises the prediction results on test set and discussion.

## Data description

To better understand the temporal effect of FFT in the Seattle area, the data set from Car2go’s API was adopted as the primary research resource in this paper. The authors developed a Java program to get access to API portal for detailed data. The collected data covered 78 days ranging from January to April in some single year (for privacy, the exact year is not stated here). The content variables received are “vehicle id,” “otime,” “dtime,” “olon,” “olat,” “dlon,” “dlat,” “distance,” “ofuel,” “dfuel,” “fuel_consumption,” “oaddress,” and “daddress.” Brief descriptions of these variables were included in Table 1.

**Table 1:** Description of booking data variables.

Name	Description
otime	Time of origin
dtime	Time of arrival at destination
olon	Longitude of origin
olat	Latitude of origin
dlon	Longitude of destination
dlat	Latitude of destination
distance	Travel distance
ofuel	Fuel level at origin
dfuel	Fuel level at destination
fuel_consumption^a^	Total consumption of fuel
oaddress	Address of origin
daddress	Address of destination

^a^The fuel consumption only refers to the number on the fuel meter

**Fig 1:**
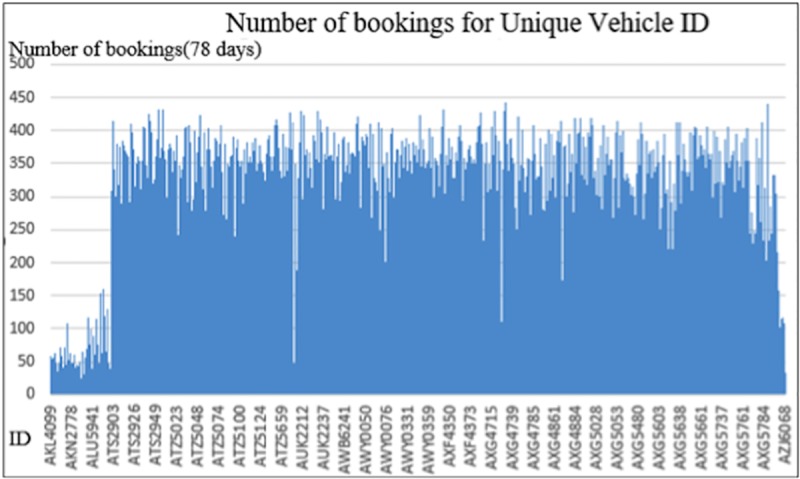
Number of bookings for unique vehicle IDs. *The vehicle IDs are not all listed in the graph because of space limitation.

After performing ID column extraction, the total amount of unique vehicle IDs in the data set is 806 (only including vehicles used during the considered), with an average of approximately 311 (range from 1 to 442) bookings counted for each vehicle in the 78 days. This means that the average daily booking count for each vehicle is approximately 4. Based on the current demand, this is not a very impressive number. A deeper insight into the daily usage is explored in Fig 1.

**Fig 2:**
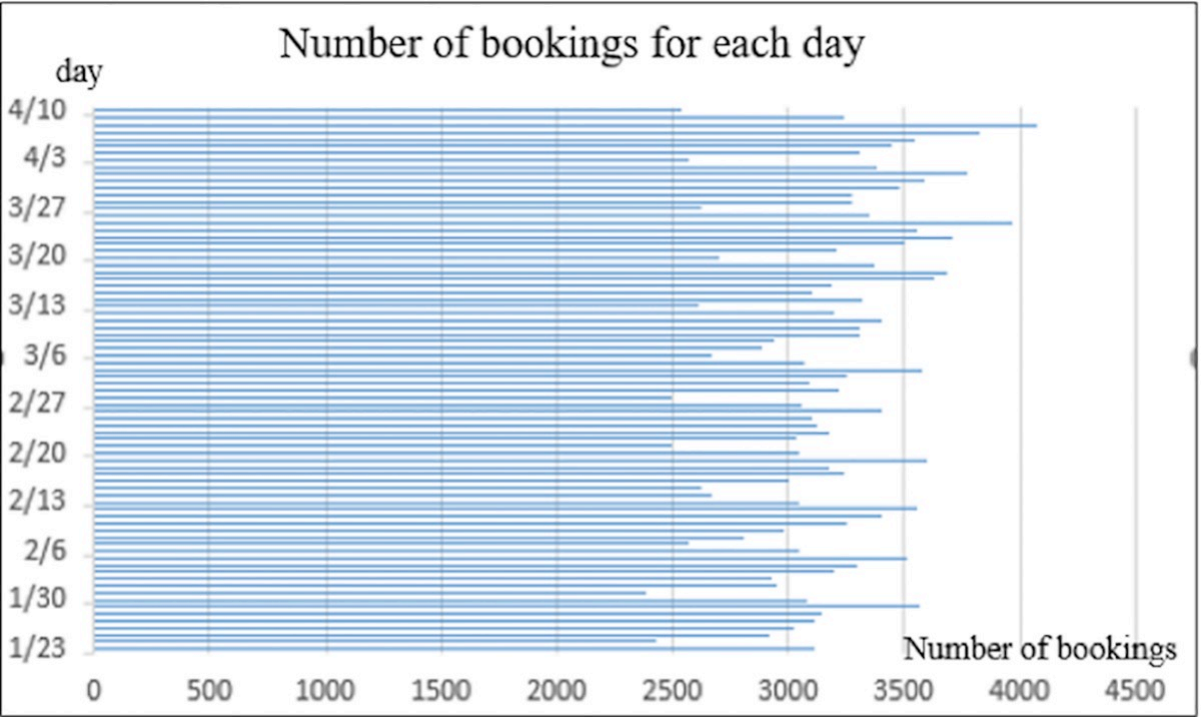
Number of bookings per day.

Then, for the daily count, a generally growing trend with time could be observed in Fig 2. The average booking count for each day was approximately 3170, ranging from 2391 to 4077, and the average booking count for each vehicle in this aspect is roughly 3.9, which is in accordance with the former conclusion. We can also see from the data and the figure, there is a clear fluctuation within each month. The booking count usually rises from the beginning of the month (roughly 2500) to the peak of over 3500 in the middle of the month and slowly decreases to the original value at the end.

**Fig 3:**
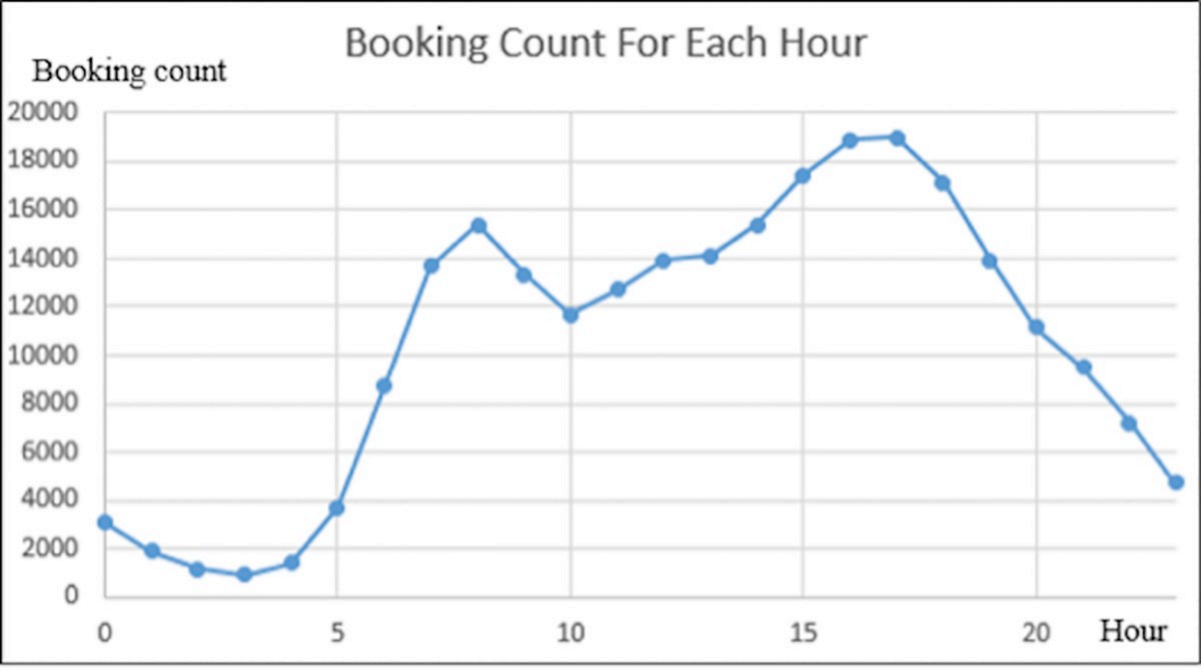
Number of bookings per hour.

To capture the short-term time pattern, based on daily data and hourly variation in booking count, a clear doublet distribution is present in Fig 3, which is consistent with the private vehicle usage distribution. The booking count involves a secondary peak at 8:00am and the maximum at 5:00pm, which coincides with the daily commute pattern. However, besides the daily commute pattern, the average booking count between 14:00 and 16:00 is higher than that of the morning peak, which shows a slight difference from either daily private vehicle usage or car2go usage. Because it is not possible to obtain detailed information of usage purpose from the data, the authors guess that such a phenomenon is associated to a non-negligible amount of recreational usage.

**Fig 4:**
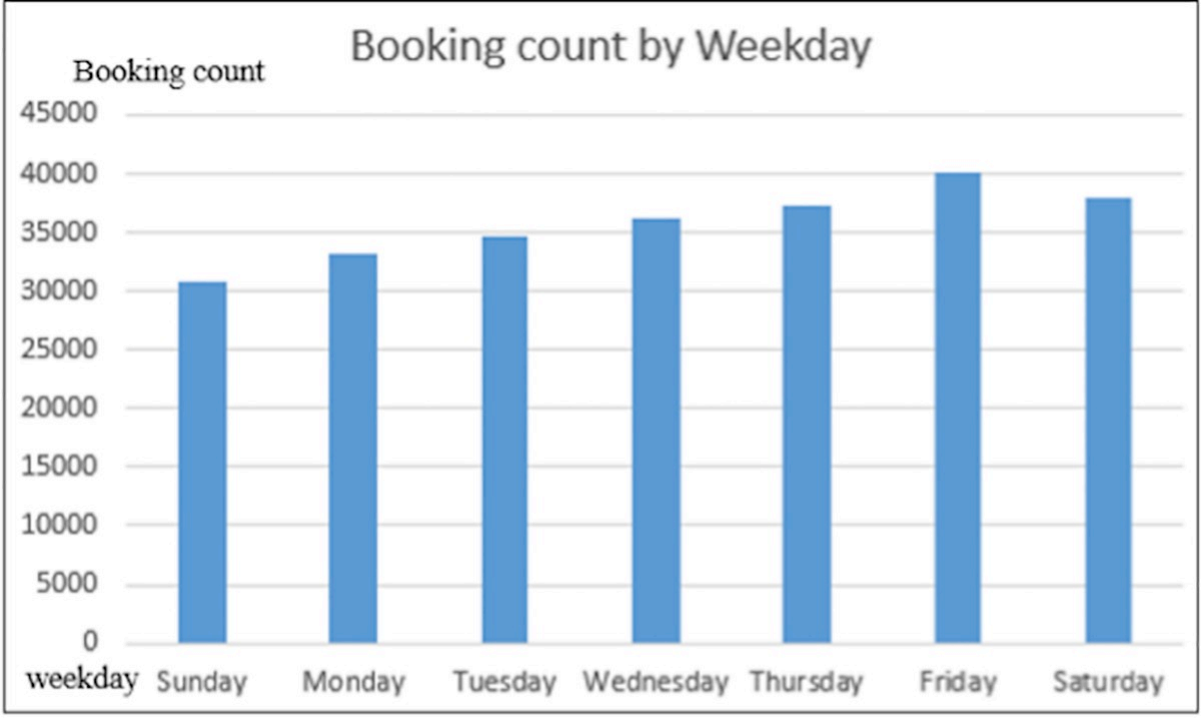
Number of bookings for weekday and weekend.

For further analysis, it is worthwhile to pay attention to daily variation as shown in Fig 4 to better capture the week pattern. Then, by extracting the statistical pattern of the result, we found that the total booking count reaches summit at Friday and drops to minimum at Sunday. Again, due to the lack in the data of detail personal travel information, it can only be inferred that there is a significant amount of recreational travel via using the car2go service because people usually go outside for fun and shopping on Fridays and Saturdays.

For the sake of spatial analysis, the entire Seattle area is divided into several zones. In general, there are primarily two ways of the division, including using the concept of the traffic analysis zone (TAZ) and using zip-code division for convenience. In this paper, the authors prefer to use the second method not only because of the difficulty in obtaining detailed information about TAZ in Seattle, but also for the purpose of connecting the booking records to the population distribution, which is related to the zip code and could be easily accessed via the Internet.

**Fig 5:**
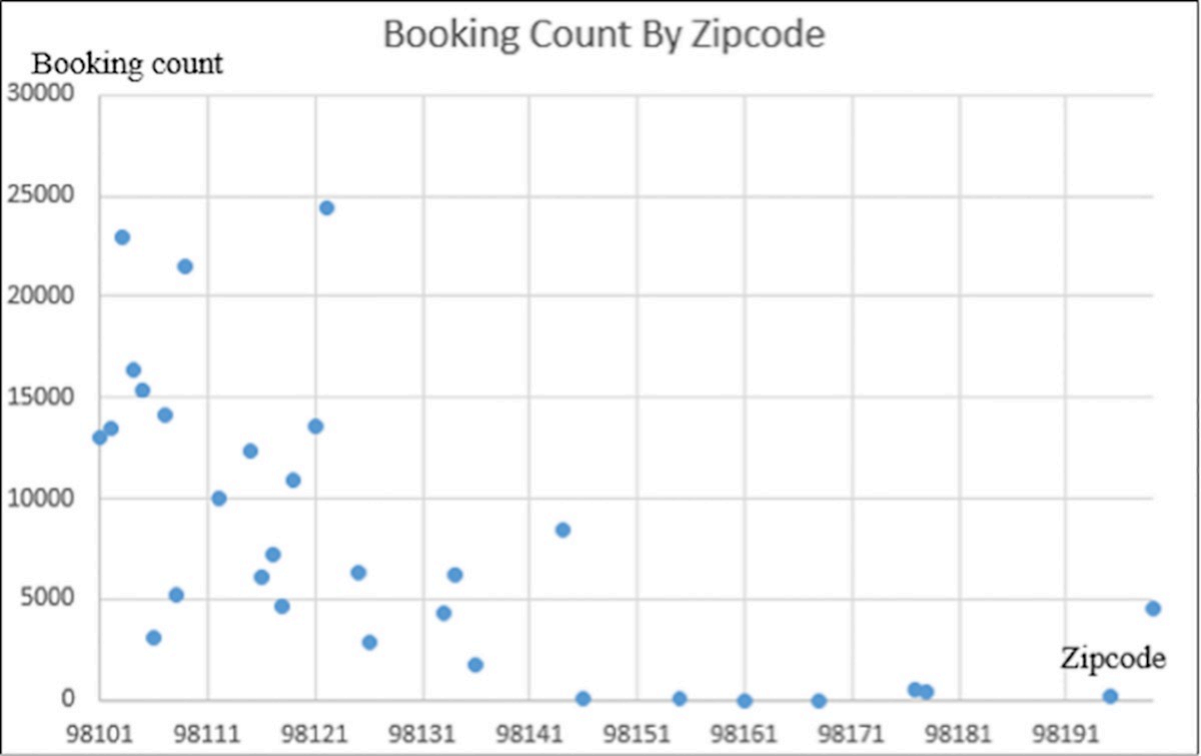
Number of bookings per zip code. *The zip codes are not all listed in the graph because of space limitation. *The zip codes are not continuous, but are discrete values.

Based on Fig 5, there is no doubt that most of the booking records lie in the areas of “U-district,” “Capitol Hill,” and “Downtown.” Most of these above-mentioned areas have far more bookings than the areas in the north and south. People in these areas need to travel for shopping, university, and other recreational purposes.

## Methodology

**Fig 6:**
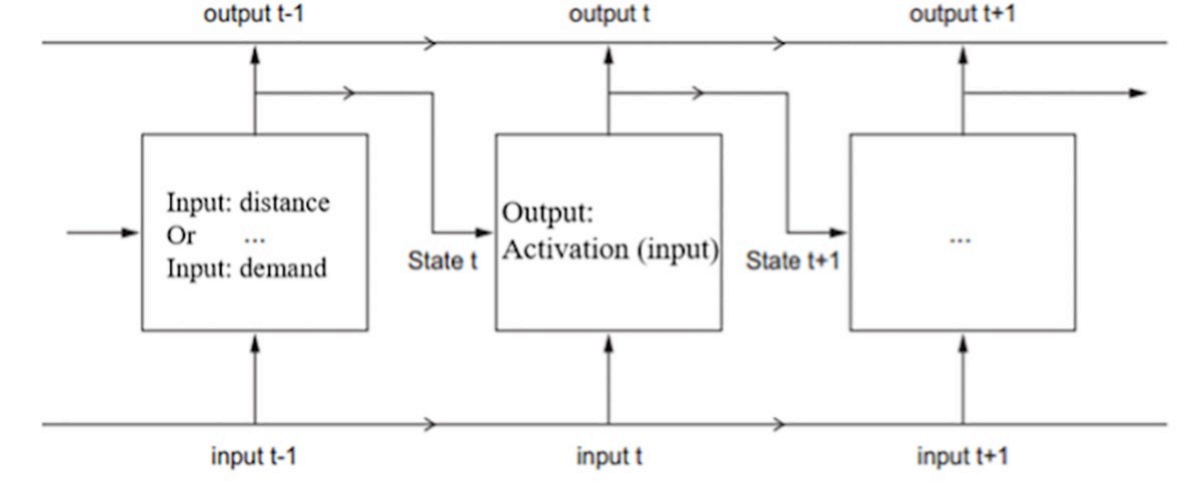
Simple recurrent neural network.

As discussed in the previous part, a comprehensive deep learning method LSTM-RNN will be analyzed to assess short-term time pattern of FFT booking demand and travel distance. The LSTM model [22–23] was derived from a simple recurrent neural network (RNN) which provides an alternative to bring information through consecutive time steps. These time steps would be used to address the short-term prediction problems. Some researchers have already concluded that in terms of traffic speed prediction, LSTM showed better performance than ARIMA and SAE and it could easily capture the hourly traffic issues like incident patterns under different road conditions [29–30]. And to improve the accuracy of short-term prediction, some refined LSTM models were proposed to solve the traffic flow predictions [31–32]. The principle of simple RNN is shown in Fig 6.

The output value for the state t is calculated through the previous cell by dot operation of two parameter matrices W and U from the previous and current state respectively. The formula is shown as follows:

$$y_{output\_t}=activation(W_{0}{output}_{t-1}+U_{0}{state}_{t})$$ (1)

Where *y*_*output_t*_ refers to the output value of state t, and *activation* means activation function works in cell t, usually, hyperbolic tangent (Tanh) and rectified linear unit (ReLU) are selected, *W*_0_ refers to a parameter matrix trained in the net which is associated with output value of cell t-1, and *U*_0_ represents a parameter matrix trained in the net which is associated with original value in the cell t.

**Fig 7:**
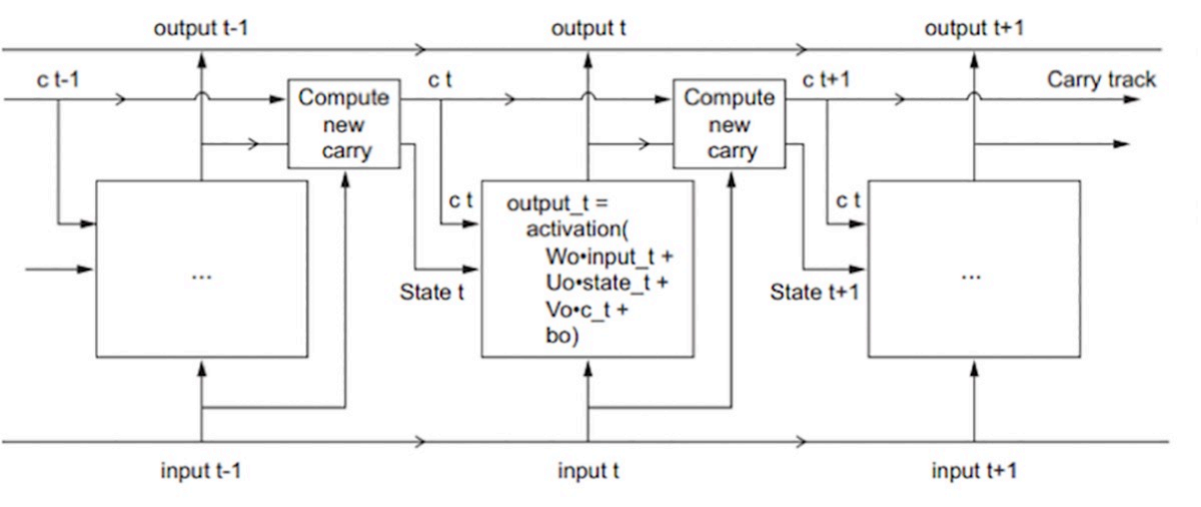
LSTM model description.

To address the problem of dropping the signals in the simple RNN, LSTM-RNN relies on establishment of a conveyor belt for the old information, which allows for storage of it to the later one, by adding parameters to form a way of removing irrelevant information and updating associated information. The new dataflow of LSTM is presented in Fig 7.

The forward propagation formulas for each cell are also given:

$$y_{output\_t}=activation(W_{0}{output}_{t-1}+U_{0}{state}_{t}+C_{t}V_{0}+b_{0})$$ (2)

$$i_{output\_t}=activation(W_{i}{output}_{t-1}+U_{i}{state}_{t}+b_{i})$$ (3)

$$f_{output\_t}=activation(W_{f}{output}_{t-1}+U_{f}{state}_{t}+b_{f})$$ (4)

$$k_{output\_t}=activation(W_{k}{output}_{t-1}+U_{k}{state}_{t}+b_{k})$$ (5)

$$C_{t+1}=i_{output\_t}+f_{output\_t}{+k}_{output\_t}$$ (6)

*C*_*t*_ is the carry parameter for state t, and is combined with input connection and recurrent connection. And the original parameter matrices *W*_0_ and *U*_0_ are decomposed into three separate sessions, which are denoted as i, f, k, and *C*_*t*+1_ for the next state could be calculated through formula 6.

The general process of an LSTM RNN model used in this paper is stated as follows:

1. Formatting data set into training and test set.
2. Data scaling. In this paper, the Min-Max Scaling method is used, Min-Max translates the feature such that it is in the given range on the training set, i.e. between zero and one. The detail mathematical equations are given as follows:

$$X_{standard}=\frac{\left(X-X_{min}\right)}{X_{max}-X_{min}}$$ (7)

$$X_{scaled}=X_{standard}*(X_{\max}-X_{min})+X_{min}$$ (8)

3 Model fine-tuning. First, the hyper-parameters inside LSTM are tested using mean squared error as the statistical indicator. Then the model itself will be compared with a simple RNN model to demonstrate its advantage.
4 Finally, a comparison research between LSTM and other statistical models such as ARIMA, SVR, single and second exponential smoothing, is conducted.

ARIMA was first introduced by Box and Jenkins in the 1970s [33], and in recent years, traffic researchers often used this model to deal with time-series problems [20–21]. The general process of an ARIMA model in this research is stated as follows:

- Observe the stationary of the raw data. If the data is not stationary, check the stability of the data after differencing.
- Define the three parameters (p, d, q) of the ARIMA model, where p stands for auto-regressive, d stands for integrated (difference), and q represents the moving average.
- Fit the model and make the prediction (usually 2 max period).

Support vector regression is extended from supported vector classification, and depends only on the subset of the training data, the cost function of which ignores any training data close to the model prediction. The general process of carrying out a SVR should be:

1. The same steps as (1) and (2) in LSTM.
2. Model fine-tuning using different kernels.

Also in order to make a comparison between LSTM-RNN and some simple statistical methods to examine the suitability of the regular data for prediction, single exponential smoothing and second exponential smoothing are adopted in this paper using the same data set.

## Results

The data set used in this paper is booking count from individual vehicle in 78 days, for the sake of short-term prediction and validation, the entire set is divided into two consecutive parts, 80% for training, and 20% for prediction (test) respectively. From each hourly short-time step, the travel demand and travel distance are involved. The sample data are provided in Table 2.

**Table 2:** Summary of sample data.

Time Step	Travel Distance (mile)	Travel Count
01D00H (train)	164	68
01D01H (train)	100	43
………………..		
62D23H (train)	…	…
63D00H (test)	…	…
………………..		
78D23H (test)	…	…

In the first column of Table 2, data collected from the first 62 days are used for training, and the rest are used for test. The second and the third column are used to tell the total distance and total booking count for the corresponding time step.

After separating the data set, the value in each cell is scaled using the Min-Max method depicted in Eqs (7) and (8).

### Travel demand prediction

**Fig 8:**
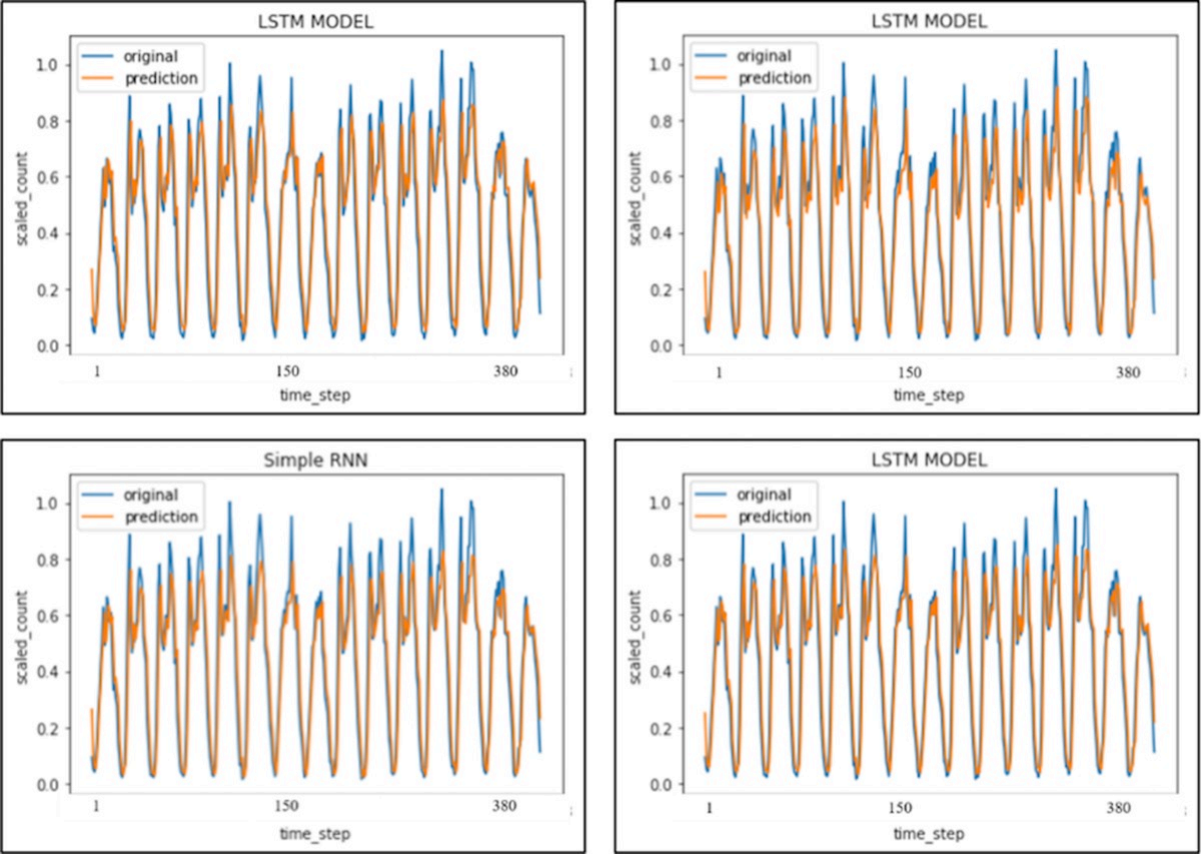
Top-left (LSTM with “relu” activation, 3 layers and 50 hidden nodes). Top-right (LSTM with “tanh” activation, 3 layers and 50 hidden nodes). Bottom-left (Simple RNN) Bottom-right (LSTM with “relu” activation, 3 layers with 20 hidden nodes).

In order to better understand short-term temporal influence on FFT travel demand, the booking records are used to analyze the trend for the near future. Several hyper-parameters in LSTM model are first tested, the results are showed in Fig 8 and Table 3.

**Table 3:** Summary of model fine-tuning.

Model	DISCRIPTION	Loss (MSE)	Adjusted R2
Simple RNN	Simple 1 layer RNN	0.0215	0.7104
LSTM RNN	3 layers 10 hidden nodes “relu” activation	0.0131	0.8034
LSTM RNN	3 layers 20 hidden nodes “relu” activation	0.0126	0.8176
LSTM RNN	3 layers 30 hidden nodes “relu” activation	0.0126	0.8154
LSTM RNN	3 layers 40 hidden nodes “relu” activation	0.0125	0.8236
LSTM RNN	3 layers 50 hidden nodes “relu” activation	0.0126	0.8212
LSTM RNN	3 layers 50 hidden nodes “tanh” activation	0.0134	0.8001

From Fig 8 and Table 3, the overall hourly periodical trend (reached peak around 8 am and 5 pm which is in accordance with the commuter pattern) is captured by prediction results from all the models, although an apparent and slight underestimation of max value occurs. The simple RNN model has some difficulty in explaining the sudden change of data, which is improved with LSTM. The prediction for the time steps involved with middle or lower booking count shows a higher precision in general. These data have superior stability compared to those in peak hours.

And the model fine-tuning results showed that those models using “relu” function exhibit better prediction for the final results compared to those using “tanh”. In addition, there is absence of sufficient proof for hidden layer nodes number, which facilitates the model accuracy. However, the model MSE does not change too much with a sharp increase of the hidden layer nodes number from 10 to 50. So, in conclusion, it is confirmed with high confidence that LSTM with a “relu” activation function combined with 30 hidden nodes number will be a good choice.

**Fig 9:**
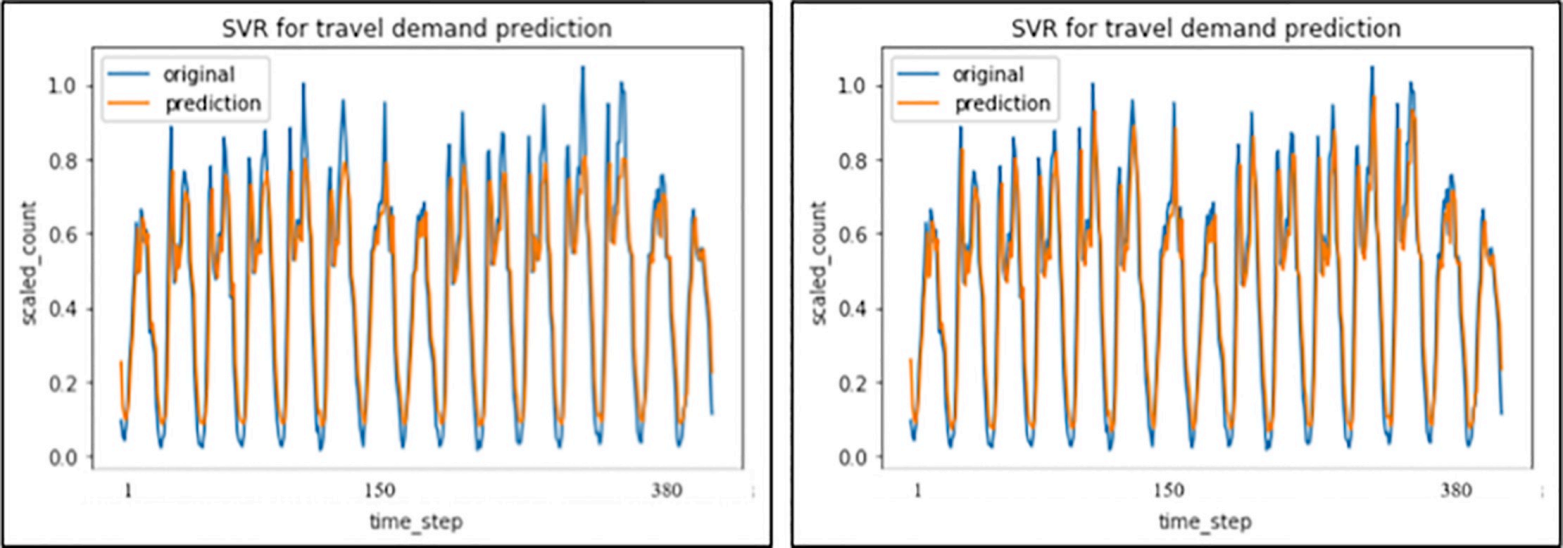
SVR with ‘rbf’ and ‘linear’ kernel prediction results.

In the following, by attempts of different combinations of p, d and q values, a satisfactory ARIMA model can be obtained. Besides, SVR (Fig 9) and two simple statistical methods are also built for comparison. The results are depicted in Table 4.

**Table 4:** Summary of ARIMA SVR and two simple statistical model fitting.

Model	R-square	Loss(MSE)
ARIMA(7, 0, 0)	0.657	0.0513
SVR with ‘rbf’ kernel	0.745	0.0417
SVR with ‘linear’ kernel	0.807	0.0125
Single exponential smoothing	…	0.2764
Second exponential smoothing	…	0.2134

In the first attempt, the author used ARIMA(7, 0, 0) to predict hourly variation of travel demand in a day, but the short-term temporal prediction results showed a worse performance compared to LSTM model. However, for SVR, the “linear” kernel model apparently outperforms the “rbf” kernel, which obviously indicates a linear hyper-plane and the performance of “Linear” kernel SVR is very close to LSTM. And both LSTM and “Linear” kernel SVR are at the same acceptable scale. And the two simple statistical methods of single exponential smoothing and second exponential smoothing provided a worst prediction results, because they could not catch the future travel demand trend well. After model fine-tuning and comparison, LSTM and SVR with “linear” kernel are superior in predicting travel demand for short-term temporal patterns. In addition, it is worth noting here that, although SVR with “linear” kernel could reach a good result as same as LSTM-RNN, its performance sometimes varies with different scale of data set [34]. thus in terms of prediction for a long-term pattern (not limit to 78 days), the LSTM-RNN might has some advantage over SVR with linear kernel.

### Travel distance prediction

**Fig 10:**
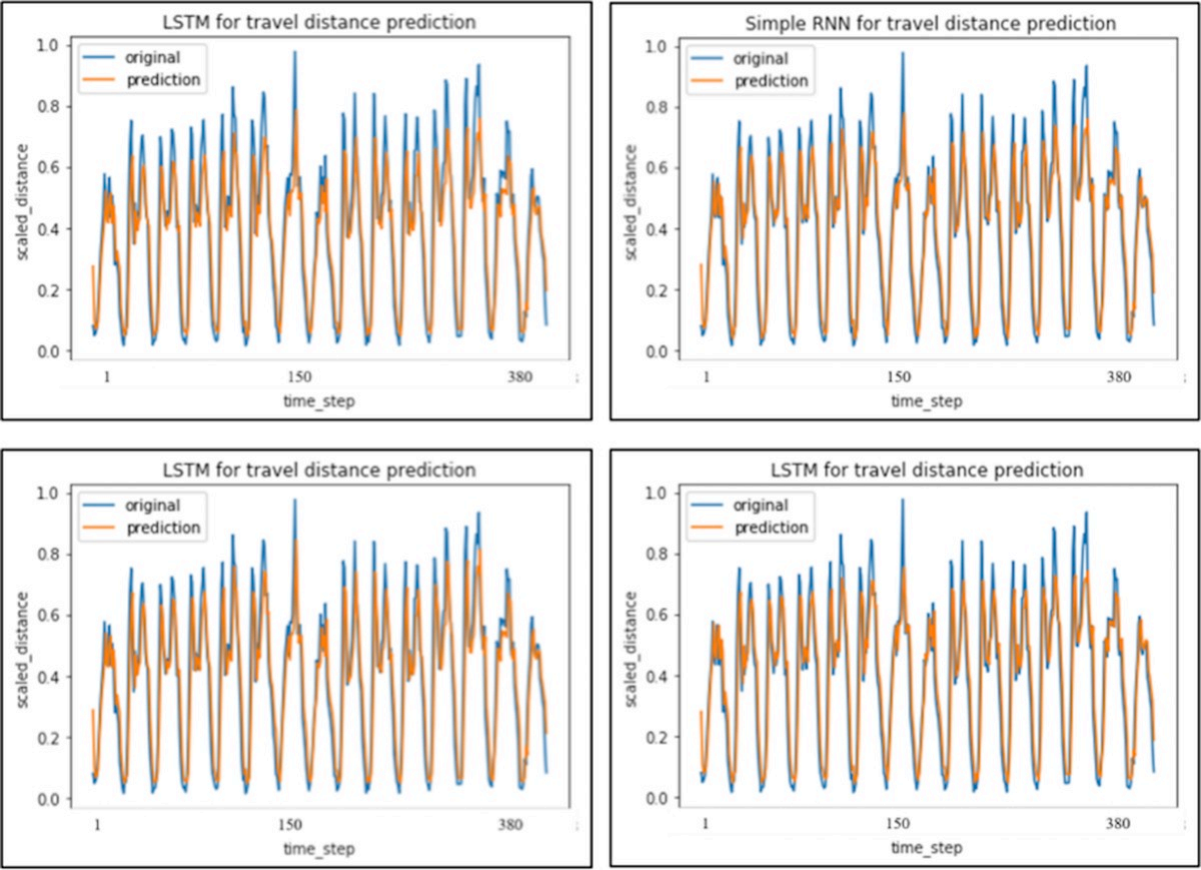
Top-left (LSTM with “relu” activation, 3 layers and 50 hidden nodes). Top-right (Simple RNN). Bottom-left (LSTM with “relu” activation, 3 layers and 30 hidden nodes). Bottom-right (LSTM with “tanh” activation, 3 layers with 50 hidden nodes).

Similarly, performance in travel demand prediction exhibits dependence on hyper-parameters in LSTM. Different hidden nodes number, activation functions and LSTM vs Simple RNN are employed to predict the test data set. The results are shown in Fig 10 and Table 5.

**Table 5:** Summary of model fine-tuning for travel distance.

Model	DISCRIPTION	Loss (MSE)	Adjusted R2
Simple RNN	Simple 1 layer RNN	0.0324	0.7096
LSTM RNN	3 layers 10 hidden nodes “relu” activation	0.0315	0.7154
LSTM RNN	3 layers 20 hidden nodes “relu” activation	0.0287	0.7438
LSTM RNN	3 layers 30 hidden nodes “relu” activation	0.0265	0.7564
LSTM RNN	3 layers 40 hidden nodes “relu” activation	0.0278	0.7536
LSTM RNN	3 layers 50 hidden nodes “relu” activation	0.0296	0.7157
LSTM RNN	3 layers 50 hidden nodes “tanh” activation	0.0307	0.7136

From Table 5 and Fig 10, the periodical trend is still present in prediction results for all the models. The model performance drop a bit compared to travel demand analysis because travel distance involved more uncertainties compared to travel demand. In such case, simple RNN could be an optional choice because there is no significant difference between MSE and adjusted R square. Prediction for those time steps corresponding to middle or lower travel distance exhibits higher precision. The predicted results in normal hours demonstrate better stability than those in peak hours, which indicate a similar short-term temporal pattern as private cars.

Similar to travel demand prediction, those models using “relu” function have slightly better batter accuracy in prediction of the final results compared to those using “tanh”. Change occurs as hidden node number came close to 30, which indicated an overfitting problem in test set. Without dropping out the parameter, a 30 hidden nodes number with “relu” activation function could be an optimal choice for predicting travel distance.

**Fig 11:**
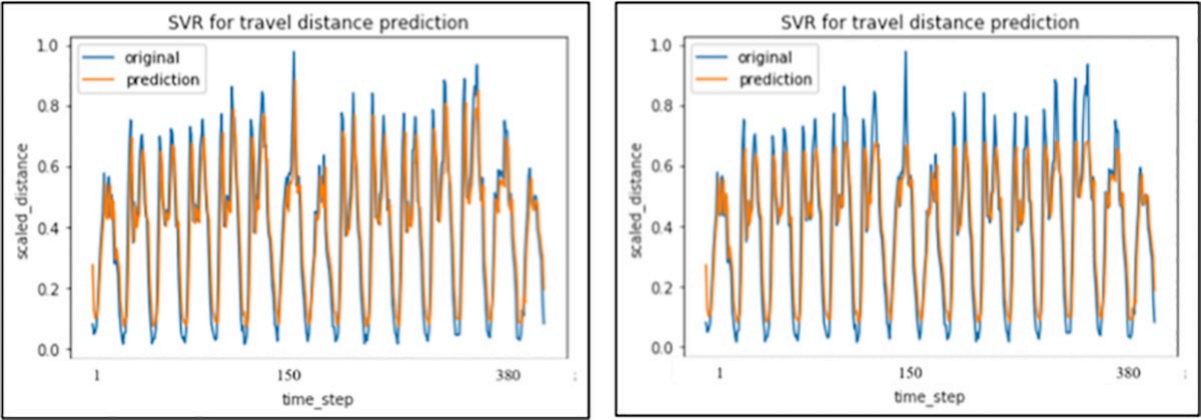
SVR with ‘linear’ and ‘rbf’ kernel prediction results.

Finally, the results of pre-refined ARIMA, SVR with different kernel functions and two simple statistical methods were also given in Table 6 and Fig 11.

**Table 6:** Summary of ARIMA, SVR and simple statistical model fitting.

Model	R-square	Loss(MSE)
ARIMA(7, 0, 6)	0.601	0.0587
SVR with ‘rbf’ kernel	0.684	0.0396
SVR with ‘linear’ kernel	0.769	0.0234
Single exponential smoothing	…	0.2687
Second exponential smoothing	…	0.1969

The ARIMA model again showed weakness in predicting travel distance, with a R-square of only 0.6. The reason is that ARIMA is more suitable for those data sets with highly inner periodical feature, and the travel distance contains much more uncertainties than the travel demand. SVR with ‘linear’ kernel could get an approximate result compared to a comprehensive LSTM, while SVR with ‘rbf’ kernel is much weaker in the prediction. And the last two simple statistical methods of single exponential smoothing and second exponential smoothing display a less favorable prediction compared to the other methodologies in this paper.

## Conclusion

In this paper, both the artificial intelligence tools and statistical methods are incorporated for hourly travel demand and travel distance prediction for free floating car sharing mode. For artificial intelligence tools, the simple recurrent neural network and long short term memory RNN are selected to train and predict the data, for comparison, the popular ARIMA model, Support Vector Regression model and two other simple statistical methods are also offered as a comparison for the accuracy and robust of LSTM. Through the research, several conclusions could be drawn as follows:

1. On average, there are approximately 4 bookings count for each vehicle per day, and hourly booking count demonstrated clearly doublet pattern, which is consistent with daily commute pattern. Moreover, according to the weekday analysis, total booking count reached summit at Friday and dropped to the minimum at Sunday. Spatial analysis showed booking count pattern is consistence with population density.
2. The rest part of this paper is focused upon offering deep learning method to provide an hourly short-term prediction of travel demand and travel distance. RNN and its refined version LSTM were offered to deal with that. The principle and structure of RNN and LSTM were given, followed by the detail steps of building the model. And the steps for comparison between SVR and ARIMA were also demonstrated in the methodology part.
3. Prediction results for travel demand shows LSTM with around 30 hidden layer nodes number and activation “relu” function outperform the other deep learning models including simple RNN, the SVR with “linear” kernel, which demonstrate the same performance as comprehensive LSTM although its performance could vary across different scale of dataset. And the ARIMA, SVR with “rbf” kernel and two other simple models performed weaker in the short-term prediction. And all models could illustrate the original periodical doublet pattern present in the raw data.
4. And the advantage of LSTM in predicting the short-term hourly variation in travel distance is not so prominent compared with other models. It produced overfitting issue while the hidden layer nodes number increased to 30, which means the Loss (MSE) started to drop as hidden layer nodes number keep increasing. Again, SVR with ‘linear’ kernel gave a good prediction results which indicate this might be a suitable alternative under condition of limited amount of data.
5. Since our first step is to predict the travel demand and travel distance for free floating car sharing, in the future, the authors prefer doing the research on demonstrating correlation between these two variables, some statistical and artificial methods will be applied [11–13].

The tools and results offered in this paper could be further applied to the next level research, for example, extracting the car trajectory information from the data set to further study human mobility and large scale demand [6–10], making a prediction of emission gas intensity caused by free-floating vehicles. And predicting short-term temporal variation in travel demand could provide a way for addressing vehicle relocating problem and traffic flow control issues with consideration of the spatial problem.

## Supporting information

S1 File Relevant data set. (CSV)
